# Systems pharmacology approaches for optimization of antiangiogenic therapies: challenges and opportunities

**DOI:** 10.3389/fphar.2015.00033

**Published:** 2015-02-20

**Authors:** Satish Sharan, Sukyung Woo

**Affiliations:** Department of Pharmaceutical Sciences, College of Pharmacy, The University of Oklahoma Health Sciences Center, Oklahoma City, OK, USA

**Keywords:** systems pharmacology, biomarkers, dose selection, antiangiogenic therapy, resistance, targeted therapies, biologically effective dose, bed

## Abstract

Targeted therapies have become an important therapeutic paradigm for multiple malignancies. The rapid development of resistance to these therapies impedes the successful management of advanced cancer. Due to the redundancy in angiogenic signaling, alternative proangiogenic factors are activated upon treatment with anti-VEGF agents. Higher doses of the agents lead to greater stimulation of compensatory proangiogenic pathways that limit the therapeutic efficacy of VEGF-targeted drugs and produce escape mechanisms for tumor. Evidence suggests that dose intensity and schedules affect the dynamics of the development of this resistance. Thus, an optimal dosing regimen is crucial to maximizing the therapeutic benefit of antiangiogenic agents and limiting treatment resistance. A systems pharmacology approach using multiscale computational modeling can facilitate a mechanistic understanding of these dynamics of angiogenic biomarkers and their impacts on tumor reduction and resistance. Herein, we discuss a systems pharmacology approach integrating the biology of VEGF-targeted therapy resistance, including circulating biomarkers, and pharmacodynamics to enable the optimization of antiangiogenic therapy for therapeutic gains.

## INTRODUCTION

Therapeutic intervention in diseases takes place within a milieu of factors, including drug pharmacokinetics, signaling pathways, mechanisms of drug action, and compensatory processes. Studying any single pathway, mechanism of action, or interactive process in isolation has limited value in improving our understanding of the complexity of disease physiology. This is evident in the redundancy of signaling networks, feedback, and cross talk between multiple regulatory processes (Moriya et al., 1996; Pawson and Warner, 2007; Logue and Morrison, 2012). A systems approach is required to quantitatively integrate underlying disease and information contributing to treatment response and resistance. Systems pharmacology combines large scale experimental studies, pharmacokinetics, mechanisms of action, signaling pathways, adaptation mechanisms, biomarker, and pharmacodynamic data in a quantitative framework utilizing computational methods. This approach can facilitate understanding of disease systems, their mechanisms of action and pathways, and hypothesis development (Agoram, 2014). Systems pharmacology further offers a tool for translational considerations from non-clinical models to patients, realizing the bench to bedside paradigm (Allerheiligen, 2010; Kreeger and Lauffenburger, 2010; van der Graaf and Benson, 2011; Demin et al., 2013; Rogers et al., 2013; Vicini and van der Graaf, 2013; Visser et al., 2014). Herein we discuss systems pharmacology approaches to achieve a mechanistic understanding of the dynamics of circulatory biomarkers for antiangiogenic agents, thereby guiding selection of doses that can maximize the therapeutic benefits.

## CHALLENGES IN ANTIANGIOGENIC THERAPIES

Angiogenesis is critical for tumor growth and metastasis. VEGF signaling is an extensively studied pathway for blocking tumor angiogenesis. Several antiangiogenic agents targeting the VEGF-pathways have been approved and are important modalities in the management of advanced cancers. Bevacizumab, a therapeutic antibody targeting VEGF and various VEGF receptor tyrosine kinase inhibitors (TKIs), have shown clinical benefit in solid tumors. However, the benefits of VEGF-targeted agents are short-lived and resistance to anti-VEGF agents rapidly emerges after an initial response phase, leading to restored tumor growth and progression. This rapid development of resistance to therapy constitutes a major clinical obstacle to providing extended therapeutic benefits with this class of drugs. Thus, effective strategies are needed to delay or prevent resistance to VEGF antiangiogenics.

Resistance to antiangiogenic agents arises through multiple mechanisms, including the activation of compensatory responses that are mediated by malignant cells and stroma cells within the microenvironment. Angiogenesis is a highly adaptive biological process. Tumors can resume angiogenesis and progress using diverse angiogenic signaling, including VEGF, FGF, HGF, PDGF, PlGF, and several proangiogenic cytokines. Numerous compensatory angiogenic factors are upregulated upon anti-VEGF therapy in a dose-dependent manner (Ebos et al., 2007), suggesting that dose intensity and frequency influence the development of therapy resistance. Higher doses of anti-VEGF therapy can create favorable conditions for metastasis by upregulating these growth factors (Ebos et al., 2009a). This emphasizes the importance of finding optimal dosing schedules for anti-VEGF therapy. The current dosing approach does not consider the best way to delay or prevent resistance to VEGF-targeted therapy, and thereby improve patient survival beyond a few months (Jubb et al., 2006; Azad et al., 2008; Cannistra, 2008).

## BIOLOGICALLY EFFECTIVE DOSES OF ANTI-VEGF THERAPY

Oncology drug development often involves the maximum-tolerated dose (MTD)-based paradigm, even when data suggest that a drug maximally inhibits its target at lower doses. The recent analysis by the U.S. Food and Drug Administration (FDA) showed that inappropriate dose selection was the major cause of post-marketing requirements for oncology drugs approved between 2011 and 2013 (Prowell, 2014). Clinically recommended doses are often derived based on their safety profiles. Toxicity has been the primary end point for conventional dose-finding strategies (Parulekar and Eisenhauer, 2004; Le Tourneau et al., 2009). Since antiangiogenic therapies are mostly cytostatic in nature, they do not always conform to the concept that MTD produces maximum benefits (Sleijfer and Wiemer, 2008). Studies have revealed better therapeutic benefits when lower doses of antiangiogenic therapies were used in combination with other treatments (Kabbinavar et al., 2003; Huang et al., 2012). Similar results were observed with other targeted therapies, such as mammalian target of rapamycin kinase inhibitor, in which a lower dose of 25 mg was selected as the recommended dose for treatment after testing 25, 75, and 250 mg doses (Atkins et al., 2004). Likewise, in a study of 24 consecutive Phase I clinical trials, in which 97.7% of participants received targeted agents, patients receiving lower (≤25% MTD) doses responded as well as those patients receiving medium (25–75% MTD) or high (≥75% MTD) doses (Jain et al., 2010). These findings support the concept that higher doses are not necessarily the most effective.

Higher doses of anti-VEGF therapies can lead to pronounced anti-vascular effects and, subsequently, hypoxia in the tumor, e.g., treatment-induced hypoxia. Treatment-induced hypoxia stimulates several compensatory biological processes to circumvent continued VEGF inhibition, leading to resistance to therapy (Harris, 2002; Casanovas et al., 2005; Drevs et al., 2005; Kerbel, 2005; Mizukami et al., 2005; Hendriksen et al., 2009; Casanovas, 2011). This excessive pruning also leads to reduced delivery of therapies into the tumor (Jain, 2005; Van der Veldt et al., 2012; Van der Veldt and Lammertsma, 2014). Tumors have abnormal vasculature, which leads to an abnormal blood supply that produces hypoxic regions in the tumor. Hypoxia has been also implicated in tumor progression by increasing genomic instability (Nelson et al., 2004) and selection of more malignant cancer stem cells with increased metastatic potential (Bottaro and Liotta, 2003; Conley et al., 2012). Therefore, antiangiogenic therapy can produce more challenges than benefits, if it is inappropriately administered (Huang et al., 2013b; Jain, 2013, 2014). This is consistent with RK Jain’s vascular normalization concept, in which the judicious use of antiangiogenic drugs can lead to more efficient delivery of drugs and oxygen to the tumor cells (Jain, 2005). Utilization of the vascular normalization strategy has been shown to improve cancer immunotherapy (Huang et al., 2012, 2013a) and survival in glioblastoma patients (Sorensen et al., 2012; Emblem et al., 2013). Therefore, there is a critical need to find the biologically effective dose (BED) that balances between normalization and excessive anti-vascular effects from antiangiogenic agents, since suboptimal and higher doses can fail to alleviate hypoxia. Further, the BED can minimize stimulation of alternative, compensatory proangiogenic signals in response to treatment-induced hypoxia, and thus limit the rapid development of treatment resistance, extending the therapeutic benefits of antiangiogenic agents (Jubb et al., 2006; Azad et al., 2008; Cannistra, 2008).

## DYNAMICS OF CIRCULATING ANGIOGENIC BIOMARKERS

The transient effects of antiangiogenic therapy predominantly result from a redundancy in the angiogenesis signaling that mediates tumor escape from anti-VEGF therapy. Many of the signaling molecules (circulating angiogenic factors, or CAF) within these compensatory pathways can be detected systemically in patients treated with VEGF-targeting agents. For example, increases in VEGF and PlGF, and decreases in VEGFR2 can be observed. These changes are considered a “class” effect of VEGF-targeted therapies (Jain et al., 2009). Many of these observed CAF changes are recapitulated in tumor-bearing mice in a dose-dependent manner, and are correlated with antitumor activity (Ebos et al., 2007). Thus, CAF are increasingly recognized as important pharmacodynamic biomarkers for better understanding the treatment response and aiding in the identification of the optimal dosing schedules for VEGF-targeted therapy (Huang et al., 2013b). Understanding the molecular interactions between therapy-induced CAFs and resistance to VEGF-targeted agents can inform the development of strategies to delay or overcome resistance to antiangiogenic therapy (Jain et al., 2009; Clarke and Hurwitz, 2013).

**FIGURE 1:**
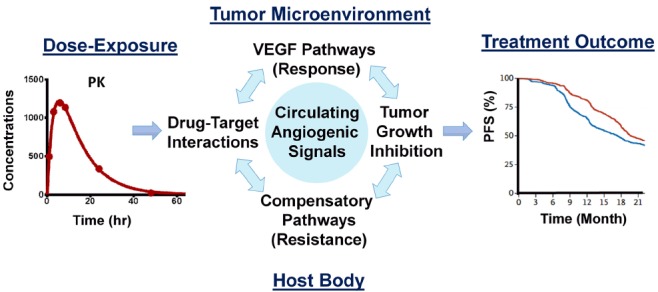
**Key components of the systems pharmacology model for anti-VEGF therapy.** The model integrates the pharmacokinetics of the drug, antitumor activity, circulating angiogenic biomarkers emanated from host and tumor cells, and therapeutic endpoints based on the drug’s response and compensatory mechanisms within a quantitative framework, to realize a bench to bedside paradigm.

These circulating biomarkers are dynamic, altered over the course of treatment by variables including *in vivo* drug concentrations (PK), changes in the tumors (e.g., antitumor effect and disease progression), the biological turnover of signaling molecules, compensatory mechanisms, tumor-independent CAF induction by normal cells in the host body, and the development of resistance. These diverse contributing factors create uncertainty when attempting to use dynamic biomarkers. Mathematical modeling can play an important role in understanding and utilizing the biomarkers to find the optimum biological dose and schedule which can delay the onset of therapy resistance (Duda et al., 2013). A recent study showcased the utility of computational models in identifying dosing schedules to manipulate the dynamics of the development of resistance to EGFR-targeted therapy (Foo et al., 2012; Dolgin, 2014). A systems pharmacology approach using multiscale computational modeling offers a tool to integrate the biology of response and resistance to VEGF-targeted therapy, including circulatory biomarkers and the pharmacokinetics/pharmacodynamics of antiangiogenic drugs (Figure 1), to optimize therapeutic gains.

## SYSTEMS PHARMACOLOGY APPROACH TO ANTIANGIOGENIC THERAPY

The major challenge in developing a systems pharmacology model is how to integrate the dynamics outside the cell (phar macokinetics) with their downstream effects in terms of protein formation or pharmacodynamic effects. PK/PD modeling has been used to explain the relationship between pharmacokinetics and the end downstream effects. What is missing is the mechanistic information in between. Limiting our investigation to antiangiogenic therapy, we anticipate three major challenges to filling this gap: (1) determining the interaction of ligands to their receptors and perturbation by drug molecules, (2) integrating the ensuing signal from these receptors with the downstream protein production machinery, and (3) accounting for interaction between various cell types, that produces pharmacodynamics responses and resistance.

### DRUG-TARGET INTERACTION

Receptor occupancy theory is well-developed and can be readily utilized to integrate this process (Black and Leff, 1983; Black et al., 1985; Mager and Jusko, 2008; Chen et al., 2009; Goodman and Redberg, 2014). We must be mindful that biology is complex and there are many subtypes of ligands, receptors, and co-receptors which have varying degrees of affinity and modulatory functions. Ligand-receptor interaction for angiogenesis involves the VEGF family of ligands (VEGF-A, B, C, D, and PlGF), three main receptors (VEGFR-1, -2, and -3), co-receptors NRP-1 and NRP-2, and heparan sulfate proteoglycans. NRP- and, -2 and proteoglycans play modulatory roles in ligand-receptor interaction; even VEGF-A is alternatively spliced to form VEGFA121, VEGFA145, VEGFA165, and VEGFA189 (Hoeben et al., 2004; Koch et al., 2011; Tugues et al., 2011). Popel and colleagues have contributed extensively to our understanding of the kinetics and interaction of VEGF ligands and receptors (Stefanini et al., 2010; Finley et al., 2011, 2013; Finley and Popel, 2012, 2013; Tan et al., 2013). Although a potential contribution of other ligand and receptor isoforms and families may be recognized, several studies have simplified these interactions by accounting for the most important VEGF ligand, VEGF-A (165), and receptor VEGFR-2 interaction as the major players in angiogenesis (Sharan and Woo, 2014; Zhang et al., 2014). The combination of competitive ligand receptor binding and an inhibitory Hill function model can be used to explain the VEGF-induced VEGFR activation and inhibitor-induced VEGFR inactivation (Sharan and Woo, 2014).

### SIGNAL TRANSDUCTION

Signaling pathways are an important component of a systems pharmacology model, which links receptor-ligand interaction to pharmacodynamic outputs (Iyengar et al., 2012). VEGF binding to its receptors led to the phosphorylation of the tyrosine kinase domain, which in turn initiated the canonical downstream signaling cascades involved in proliferation, migration, survival, and permeability (Tugues et al., 2011). Ordinary differential equation (ODE)-based models, also termed mechanistic or physicochemical models (Birtwistle et al., 2013; Zhang et al., 2014), are often used to describe the canonical signaling cascades. The advantage of this approach being more mechanistic can help a personalized medicine paradigm by incorporating information related to genomic variation and mutation (Iyengar et al., 2012). The limitation of this approach is the currently incomplete mechanistic knowledge of several mediators, signaling processes, and parameter identifiability. Given the incomplete mechanistic knowledge of several mediators and signaling processes, or in the absence of measurement of mediator signaling molecules, a more empirical quantitative logic (QL; Kirouac et al., 2013; Kirouac and Onsum, 2013) or transduction model (Mager and Jusko, 2001) can be utilized to characterize signal transduction. The QL approach has been elegantly explained by Kirouac and Onsum (2013) in building multiscale models which capture the features of oncogenic signaling networks. The transduction model has the flexibility of handling multiscale events with different transit time parameters to account for time needed for signal transduction from receptor-ligand interaction to nucleus, time for cell machinery to form proteins, and to show the pharmacodynamic effects on tumor growth. It is vital to take a balanced approach between mechanistic representation of the signaling pathway and the model’s predictive power (Sharan and Woo, 2014).

### THERAPEUTIC AND COMPENSATORY RESPONSES TO ANTI-VEGF THERAPY

Ideally, signaling events are linked to tumor growth kinetics. Tumor inhibitory effects of anti-VEGF agents can be described by adapting well-established models (Simeoni et al., 2004, 2013; Ribba et al., 2014). In addition to tumor growth inhibition, systemically quantifiable biomarkers such as CAFs can serve as an important measurement to identify disease progression, make dose selections, or stratify patients. Indirect response models can be effectively used to capture inhibition, stimulation, and turnover rates of biomarkers modulation (Mager et al., 2003). We can use non-linear feedback regulation to account for compensatory increases in circulatory biomarkers in response to treatment-induced hypoxia by anti-VEGF agents.

The contributions of host cells and stroma cells within the tumor microenvironment have been increasingly recognized to play an important role in cancer progression and treatment (Ebos et al., 2007, 2009b; Kerbel and Ebos, 2010; Jain, 2013; Stroh et al., 2014). This should be explored using a systems pharmacology model. Antiangiogenic therapies have been shown to upregulate various growth factors in healthy cells and are dose-dependent in non-tumor-bearing mice (Ebos et al., 2007). This dose-dependency is also observed in healthy human volunteers (Lindauer et al., 2010). Thus, it is important to characterize tumor and host cell contributions to CAF modulation and to provide mechanistic information for interpreting biomarker data in respect to antiangiogenic treatments.

## APPLICATION OF A SYSTEMS PHARMACOLOGY MODEL OF CAFs FOR DOSE OPTIMIZATION

We have recently developed a systems pharmacology model that uses sunitinib as the test drug to quantify the link between *in vivo* drug concentrations (PK), target–drug interactions, the biological target pathway, antitumor activity, and compensatory signals leading to treatment resistance (Figure 1). We used the most frequently studied CAFs, including VEGF, PlGF, and sVEGFR2. Our model predictions were consistent with the time- and dose-dependent changes in these hypoxia-derived CAFs following sunitinib given to mice at various dosages (Ebos et al., 2007). We then tested our model within a clinical setting to explain VEGF changes in patients with cancer who experience different treatment outcomes; we found that our predictions were consistent with the observed VEGF changes in patients receiving sunitinib for the treatment of metastatic renal cancer (Kontovinis et al., 2009). The stimulation/inhibition capacity and the hill coefficients of VEGF, PlGF, and sVEGFR2 in mice were similar to those reported in humans, indicating that system-specific parameters for conserved physiological processes such as angiogenesis are comparable across species (Sharan and Woo, 2014).

**FIGURE 2:**
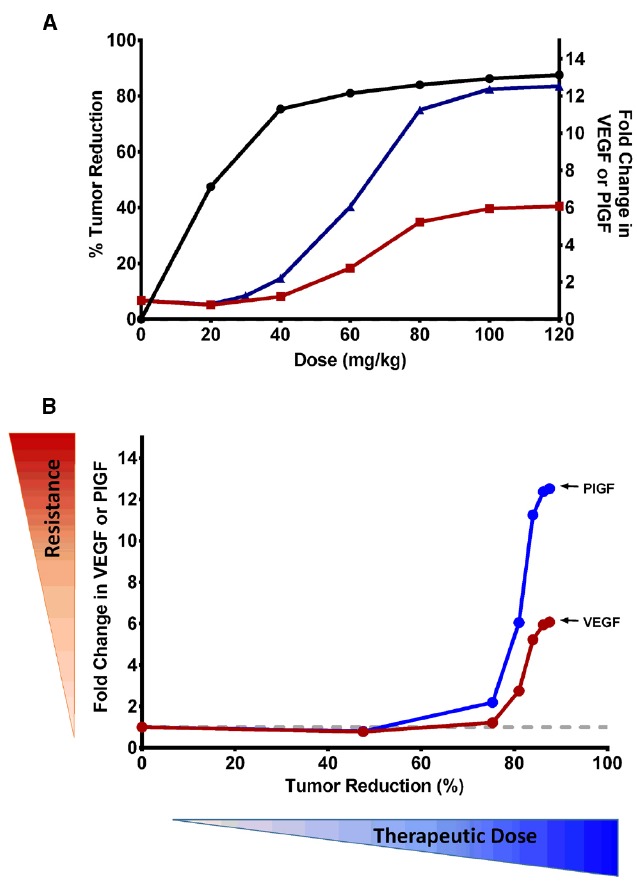
**(A)** Relationships of therapeutic efficacy and modulation of VEGF and PlGF biomarkers to sunitinib doses. Percentage reduction in tumor volume (•) and fold change in PlGF (▴) and VEGF (▪) are shown at various doses of sunitinib at the end of study. At the dose of 40 mg/kg/day, ∼75% of tumor volume was reduced, with minimal upregulation of hypoxia-dependent CAF. Further dose escalation resulted in marginal therapeutic gain (<5%), but significant upregulation of CAF, which may indicate excessive anti-vascular effects. **(B)** Utilization of CAF biomarkers in the selection of biological dose of antiangiogenic drugs. The fold change in VEGF and PlGF may serve as a surrogate marker for excessive anti-vascular effects and, in turn, potential for emerging resistance. This illustrates how the biologically effective dose may be selected in a manner which does not invoke significant hypoxia and involves little stimulation of hypoxia-dependent CAF. Monitoring multiple CAFs will be advantageous, as each factor has a different dynamic range. PlGF has a wider dynamic range than VEGF, and results in higher fold change at the same dose. This provides an advantage over VEGF, because PlGF changes are more likely detectable even at lower doses.

Our model allows us to delineate CAF changes in the tumor microenvironment and host body during VEGF-targeted therapy and to assess their impacts on tumor response and resistance to therapy. This provided insight into the possible ways to utilize the CAF for better dose guidance of these therapies, either alone or in combination. We found a relationship of tumor reduction and compensatory increase in VEGF and PlGF with increasing sunitinib doses in xenograft mice (Figure 2A). The increase in proangiogenic factors was directly related to the dose, suggesting that these CAF can be used as biomarkers to determine the optimal dose for antiangiogenic drugs. For example, in an A431 xenograft mouse model, the maximum benefit from sunitinib treatment may be achieved at a dose of 20–40 mg/kg/day of sunitinib, as such doses produce no significant changes in VEGF or PlGF levels. Further dose escalation resulted in marginal therapeutic gain (<5%), but significant upregulation of hypoxia-dependent CAF, which may indicate excessive anti-vascular effects. As such, these CAF could be used to construct a therapeutic index for antiangiogenic agents. Figure 2B illustrates this CAF biomarker-based paradigm for dose selections in particular, balancing between antitumor effects and CAF changes. The CAF modulation may serve as a surrogate marker reflecting the anti-vascular effects of antiangiogenic treatment. Ligands of tyrosine kinase receptors have been found to confer resistance by engaging survival signals redundant to those of targeted kinase (Wilson et al., 2012). If we assume that higher changes in compensatory signals are associated with higher likelihood of early onset of resistance, antiangiogenic doses may be increased up to the level at which the CAF increase from their baseline is minimal (e.g., <2-fold for VEGF).

Increasing sunitinib doses also led to VEGF and PlGF stimulation with different magnitudes (Figure 2B). This differential stimulation of pro-angiogenic factors can be exploited to facilitate dose finding. Given the inter-individual variability and heterogeneity in tumor response, monitoring multiple biomarkers, rather than relying on a single marker, would be advantageous. We found that PlGF changes were ∼2-fold higher than VEGF changes at the same dose (Sharan and Woo, 2014). This finding suggests that PlGF has a wider dynamic range than VEGF, and can ensure better detection of its change even at lower doses. Thus, monitoring PlGF and VEGF can aid in ensuring that the therapy does not fall below the minimum effective dose nor go above the excessive anti-vasculature dose. This finding is consistent with the recent study in which increased PlGF, but not VEGF, was associated with patients responding to cediranib (Batchelor et al., 2013). While we illustrated the CAF-based dose-finding strategy using VEGF and PlGF, other CAF could be used, as different tumor types and drug targets can invoke different CAF dynamics. Many concepts and the mathematical framework are broadly applicable among several tumor types and different antiangiogenic agents, and could serve as a paradigm for determining the optimal dose of targeted therapies.

Antiangiogenic therapies are often administered in combination with chemotherapy. There is increasing interest in combining antiangiogenics with other targeted therapies in order to improve therapeutic outcomes. However, since the clinical doses of many targeted therapies are determined based on MTD rather than BED, we cannot easily deduce the dosage and schedules of combinations from single agent studies. When antiangiogenic therapies are combined with drugs of same class, excessive overlapping toxicities have resulted (Azad et al., 2008, 2009). In addition, antiangiogenic therapies at higher doses could reduce the efficacy of concomitant cytotoxic agents, most likely due to reduced drug delivery by excessive vessel pruning (Van der Veldt et al., 2012). The CAF biomarker-based approach could also be useful for determining the optimal dose of combination therapy. In combination therapy, the role of antiangiogenic drugs may be focused on vascular normalization. Other therapeutics can be targeted toward killing tumor cells. In such cases, it is desirable for antiangiogenic drugs to be administered at lower doses at which the stimulation of compensatory signaling is minimal.

## CONCLUSION

Therapy-induced CAF can be effectively utilized as pharmacodynamic biomarkers to find the optimal biological dose for antiangiogenic drugs. This will ensure that the therapy maintains minimum effective dose levels, without invoking much compensatory response from the system. Routine incorporation of biomarkers into future clinical trials will be critical for the optimization of anti-VEGF agents and development of next generation of antiangiogenic regimens. Biomarker studies can be augmented by imaging studies, such as dynamic contrast-enhanced MRI (DCE-MRI), or other imaging techniques to monitor vessel integrity, permeability of blood vessels, and tumor perfusion (Murukesh et al., 2010). Thus, future strategies will require circulating biomarkers and imaging with an integrated multi-scale computational tool to guide optimal dose selection for antiangiogenic agents. As more data become available in the future, with the advance of high throughput methods like genomic data, the main challenge will be vertically integrating those data. Systems pharmacology will offer a tool to vertically integrate knowledge from pharmacokinetics, mechanisms of action, genomics, biomarkers, toxicokinetics, and pharmacodynamics. This approach yields an informed perspective from which we can streamline drug discovery and development. The knowledge gained from this approach can provide an in-depth understanding and, hence, a better approach for achieving enduring therapeutic benefits from antiangiogenic therapy.

### Conflict of Interest Statement

The authors declare that the research was conducted in the absence of any commercial or financial relationships that could be construed as a potential conflict of interest.
